# Feasibility of Muscle Synergy Outcomes in Clinics, Robotics, and Sports: A Systematic Review

**DOI:** 10.1155/2018/3934698

**Published:** 2018-04-01

**Authors:** Juri Taborri, Valentina Agostini, Panagiotis K. Artemiadis, Marco Ghislieri, Daniel A. Jacobs, Jinsook Roh, Stefano Rossi

**Affiliations:** ^1^Department of Economics, Engineering, Society and Business Organization, University of Tuscia, Viterbo, Italy; ^2^Department of Electronics and Telecommunications, Politecnico di Torino, Torino, Italy; ^3^Department of Mechanical and Aerospace Engineering, Arizona State University, Tempe, AZ, USA; ^4^Department of Mechanical Engineering, College of Engineering, Temple University, Philadelphia, PA, USA; ^5^Department of Kinesiology, College of Public Health, Temple University, Philadelphia, PA, USA; ^6^Department of Physical Medicine and Rehabilitation, Northwestern University, Chicago, IL, USA

## Abstract

In the last years, several studies have been focused on understanding how the central nervous system controls muscles to perform a specific motor task. Although it still remains an open question, muscle synergies have come to be an appealing theory to explain the modular organization of the central nervous system. Even though the neural encoding of muscle synergies remains controversial, a large number of papers demonstrated that muscle synergies are robust across different tested conditions, which are within a day, between days, within a single subject, and between subjects that have similar demographic characteristics. Thus, muscle synergy theory has been largely used in several research fields, such as clinics, robotics, and sports. The present systematical review aims at providing an overview on the applications of muscle synergy theory in clinics, robotics, and sports; in particular, the review is focused on the papers that provide tangible information for (i) diagnosis or pathology assessment in clinics, (ii) robot-control design in robotics, and (iii) athletes' performance assessment or training guidelines in sports.

## 1. Introduction

Motor tasks are achieved by activating an appropriate set of muscles [1]. The number of recruited muscles depends on the task constraints of a specific movement [1]. The central nervous system (CNS) plays the main role in the motor control since it activates the specific muscle choosing both the magnitude and the timing of the activation [2].

The organization of the CNS in controlling and coordinating a large number of muscles still represents unclearly in the field of motor control [3]. The CNS may decrease the complexity of motor control by reducing the dimensionality of the controlled variables, which is the number of muscles [4]. Since Bernstein proposed the topic of many degrees of freedom in motor control (1967), several researchers focused on understanding how the CNS can limit the control signals to activate a large number of muscles [5]. The activation of muscle synergies, rather than each muscle individually, represents the most appealing idea to explain how the CNS bypasses the difficulty in controlling a large variety of muscles [6–9]. These modules are commonly named muscle synergies, which are defined as the coherent activation, in space and time, of a group of muscles. Muscle synergies can be extracted by factorizing data acquired by means of surface electromyography (EMG). Nonnegative matrix factorization [10] is the most widespread algorithm used for factorization, even though it was demonstrated that similar results can be obtained by applying other methods, such as principal or independent component analysis and inverse Gaussian [11, 12]. Factorization leads to the computation of muscle synergy vectors (*W*) and temporal activation patterns (*C*); the first one is a time-invariant matrix that contains the weights corresponding to the contribution of each muscle to the specific synergy, while the second one is a time-variant waveform that represents the neural signal modulating the excitation of the specific synergy. EMG data can be obtained as a linear combination of muscle synergy vectors and temporal activation patterns, whose dimensions depend on the number of muscle synergies needed to perform a specific motor task [13].

In order to demonstrate that muscle synergies can objectively represent an effective tool to understand the organization of the CNS in motor control and they are not a mere output of a mathematical approach, several studies have been conducted on all the methodological issues related to EMG factorization by means of muscle synergies. In particular, the studies have been focused on the effects of the number and the selected muscles [14], the factorization algorithms [12], the averaging or concatenating repetitions of the same task [15], and the repeatability of the methods [11, 16] on the output of a muscle synergy model. All the mentioned papers demonstrated the good reliability and repeatability of the muscle synergy theory regardless of the selected muscles, the applied factorization algorithms, and the used postprocessing procedures.

Due to the promising results, muscle synergies were used to describe the muscle activity in several applications, such as clinics [6, 17–51], robotics [52–67], and sports [68–87].

The present systematic review aims at providing an overview on the possible applications of the muscle synergy theory in the three above-mentioned research fields. In particular, literature was reviewed focusing on the aims, enrolled subjects, recorded muscles, data processing, data analysis, results, and related tangible conclusions of each study.

## 2. Materials and Methods

### 2.1. Search Strategy

A literature review was performed on Scopus and PubMed databases in order to find a paper focused on the applications of the muscle synergy theory in clinics, robotics, and sports. The electronic search started on August 2017, and it finished on October 2017. The included keywords were *muscle synergies; electromyography; muscle activity; muscle coordination; EMG, muscle synergy vectors; muscle synergy activation patterns; EMG factorization; motor control; locomotion; clinical gait analysis; balance; postural control; rehabilitation; EMG-based robot control*; *robotics; sports; sports performance*. In addition, wildcard symbols, such as hyphens or inverted commas, were used to consider all possible variations of root words. Moreover, references of each found article were carefully checked not to miss important papers that have not been highlighted in the electronic search [88]. All authors conducted the literature search.

### 2.2. Inclusion Criteria

The initial inclusion criteria were based on the contents of the title and abstract. Then, the articles were screened to check whether they met the following criteria. Firstly, they were written in English; secondly, they were published from January 2006 to October 2017. Finally, we included only one paper published by the same authors if other papers and/or conference proceedings presented the same contents.

### 2.3. Data Extraction

Publications that met the initial inclusion criteria were downloaded into Mendeley Desktop 1.17.11 for further screening. In order to make the review readable and focused on the authors' intention, as claimed in the Introduction section, the included papers were firstly divided into one of the three categories: clinics, robotics, and sports. Then, the following information was collected from each paper: (i) what the aims were, (ii) what the actual application was, (iii) which subjects were enrolled, (iv) which muscles were recorded, (v) how data were processed and analyzed, and (vi) which results and conclusions were obtained.

### 2.4. Quality Assessment

To make an initial selection of the large number of papers that are available for the present review, a quality assessment was performed. The quality of each paper was assessed in terms of internal, statistical, and external validity [89]. In particular, the internal validity allows assessing the validity on bias of the research design and the operational study, the statistical validity permits quantifying statistical significance of the results, and the external validity is useful for examining the generalization of the study [90]. All the authors were asked to answer an 18-item checklist. In particular, the used checklist (Table 1) provided information on (i) internal validity (question numbers 1, 3, 4, 6, 7, 9, 12, 13, and 14), (ii) statistical validity (question numbers 15, 16, 17, and 18), and (iii) external validity (question numbers 2, 3, 5, 6, 8, 10, and 11). Each item of the checklist had to be answered with “+” or “−” corresponding to a score of 1 and 0, respectively. It is worth noticing that the 18-item checklist is similar to the ones commonly used in literature for systematical and/or meta-analysis reviews [91–95], and it was adjusted based on the specific review topic.

Papers that reached a score equal or more than 11, that is, at least 60% of “+” among the answers, in the majority of the authors can be defined as “high quality” [92, 94].

In the clinics section, a further selection of the available papers was performed taking into consideration only publications that provided tangible results useful for improving neuromuscular diagnosis and rehabilitation assessment for locomotion, balance, and upper limb functions.

Taking into account the robotics section, only papers that proposed the synergy-based control as a viable alternative to the traditional myoelectric control and that tested the synergy-based control on robotic devices with subjects were deeply discussed.

Focusing on sports, we further skimmed the paper by selecting only ones that proposed tangible outcomes useful for improving the athlete performance assessment and/or to plan an athlete-specific training session.

**Figure 1:**
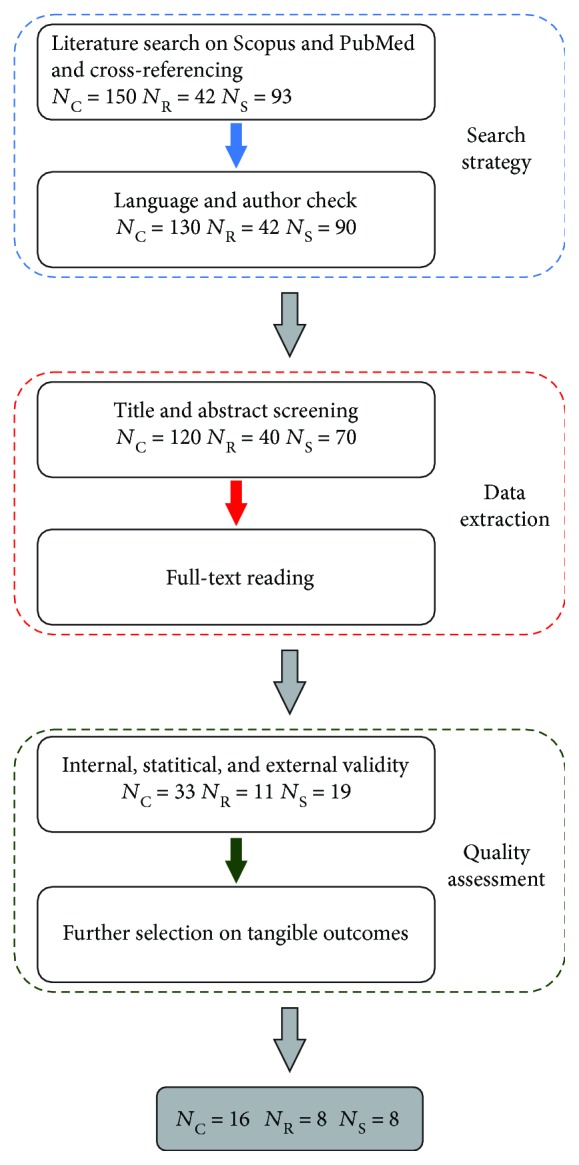
Flowchart of the systematic review process. *N*_C_, *N*_R_, and *N*_S_ stand for number of papers in the clinics, robotics, and sports sections, respectively.

A flowchart of the systematic review process is reported in Figure 1.

Since the acronyms used for the muscles were not consistent in the examined papers, we decided to standardize the name of muscles as reported in Table 2.

## 3. Results and Discussions

### 3.1. Clinics

Due to the large amount of papers focused on muscle synergies in clinical applications, this paragraph is divided into two main sections. The former takes into account the muscle synergy extraction in locomotion and balance, and the latter concerns the functionalities of the upper limb.

#### 3.1.1. Locomotion and Balance

The electronic search in the cited databases and the cross-referencing evaluation returned as a result 60 publications dealing with muscle synergies in the context of neuromuscular pathologies and rehabilitation. The application of the above-mentioned inclusion criteria allowed the selection of 17 papers that provide tangible results and a better understanding of the disabilities that result from an impaired nervous system.  Table 3 describes the distribution of the selected papers considering different pathologies that could affect locomotion and balance.

Details of all cited papers are reported in Table 4.

In the following subparagraphs, one paper on poststroke patients, one on spinal cord injury, one on Parkinson's disease, and one on cerebral palsy will be further discussed since they provided tangible results useful for improving neuromuscular diagnosis and rehabilitation assessment.

*(1) Stroke*. In this section, we report the work of Clark et al. [17], since it deeply investigated muscle synergies in poststroke subjects during walking at different speeds. The authors compared 55 subjects with poststroke hemiparesis and 20 healthy adults in terms of motor coordination during treadmill walking at different speeds. Volunteers with hemiparesis performed walking trials of 30 s duration at self-selected comfortable speed (SS) and at fastest comfortable speed (FS), while healthy participants walked at SS, FS, and six additional speeds ranging from 0.3 to 1.8 m/s. Bipolar electrodes were used to record EMGs from 8 lower limb muscles (TA, SOL, GM, VM, RF, SEMT-SM, BF_lh_, and GLU_med_) of each side. The authors found a preservation of the low-dimensional modular organization of muscle coordination in both healthy and poststroke subjects with hemiparesis, but the ability to activate independently the motor synergies was compromised in the paretic side of poststroke patients. The number of muscle synergies in poststroke individuals was reduced in the paretic side due to a merging of motor synergies, revealing impairments in lower-limb muscle coordination and an overall reduction in the complexity of locomotor control. This decrease in the number of independent synergies was also correlated with poorer walking performance, such as self-selected walking speed, speed modulation, and step length asymmetry. Thus, authors suggested that motor synergies could provide a quantitative description of the motor control deficits following stroke.

*(2) Cerebral Palsy*. In this section, we reported the work of Steele et al. [25] which examined motor synergy modifications in individuals with CP during gait. The aim of the study was to evaluate if participants with CP demonstrate lower complexity of motor coordination during locomotion compared to healthy subjects. Muscle synergies during gait were analyzed for 549 participants with CP and 84 unimpaired individuals, selected from a database of over 6600 subjects. Surface EMG signals from 5 lower limb muscles (RF, SEMT-SM, BF_lh_, TA, and GM) were acquired from the most affected side during one randomly selected barefoot cycle. Results demonstrated that fewer synergies were required to describe muscle coordination during locomotion in subjects with CP. Patients' motor control was characterized by one or two muscle synergies, while three or four synergies were required in healthy subjects. In conclusion, the outcomes allow considering the synergy number as an index for the assessment of CP.

*(3) Spinal Cord Injury*. The article reviewed in this section provides a detailed description of the effects of the incomplete spinal cord injury (iSCI) on the locomotor control of children during different locomotion tasks. Fox et al. [28] focused their work on neuromuscular control of locomotor tasks in children with iSCI and in uninjured children. Authors enrolled five children with iSCI and five age-matched controls who were asked to perform different locomotor tasks, such as treadmill walking, overground walking, pedalling, supine lower extremity flexion/extension, stair climbing, and crawling. For each task, EMGs related to both lower limbs were recorded from 6 muscles: TA, GM, VM, RF, SM-SEMT, and GLU_med_. In the assessment of altered muscle coordination, fewer motor synergies were required to explain muscle activations in the lower limbs of injured children compared to healthy ones. However, in both groups, a similar modular organization across different locomotor tasks suggested that the nervous system could coordinate complex lower limb movements starting from a small set of elementary synergies. Thus, these findings suggest that the rehabilitation of the locomotion might have benefits for a large variety of locomotor tasks.

*(4) Parkinson's Disease*. In the following, we discuss the work of Allen et al. [30], since it provides useful information about changes in muscle coordination after rehabilitation in subjects with (PD). The aim of the study was to use muscle synergies to assess changes in neuromuscular control of gait and balance in subjects with PD after a partnered, dance-based rehabilitation session. Nine volunteers affected by PD were enrolled in the study. Each subject performed an intensive rehabilitation during 15 Adapted Tango (AT) lessons with a dance instructor. The EMG signals from 13 muscles of the right leg and lower back (RA, EO, ESL, GLU_med_, TFL, BF_lh_, RF, VM, GM, GL, SOL, PL, and TA) were acquired during overground walking at self-selected speed (gait control) and during ramp-and-hold translations of the support surface (balance control). The results revealed no increase in the number of motor synergies despite improvements in gait and postural control immediately after rehabilitation. After AT, the great majority of participants showed decreased motor synergy variability and increased motor synergy consistency in both walking and balance tasks. Furthermore, this study demonstrated that the metrics of consistency, distinctness, and generalizability of motor synergies are more sensitive to improvements in gait and balance function than the number of muscle synergies.

#### 3.1.2. Upper Extremity Functions

Using the search criteria of Section 2.1, we found 60 publications referencing muscle synergies in the result of upper-limb pathology and rehabilitation training. Applying the inclusion criteria, we selected 16 papers for inclusion in this section. The most common exclusion applied to the selected papers was the lack of a clinical subject population. We made an exception by including an experimentally induced pain along with chronic pain.  Table 5 describes the distribution of the selected papers considering different pathologies that could affect the upper limb.

Details of all cited papers are reported in Table 6.

*(1) Stroke*. We found 9 papers that quantified the abnormalities in muscle coordination, evaluated the effects of therapeutic methods on the modularity of muscle coordination, and examined the neuroanatomical correlates of muscle synergies identified in the upper extremity poststroke. Among them, we reviewed the two following articles, Cheung et al. [36] and Roh et al. [38], considering that both works rigorously characterized abnormal muscle coordination in stroke using different behavioural paradigms. In addition, we reviewed Hesam-Sariati et al. [42] which examined the effects of therapeutic activities on the organization of muscle synergy as well as Godlove et al. [43] which addressed the neural correlates of normal and abnormal muscle synergies identified from a unique stroke case.

*(2) Stroke: Quantification of Abnormalities in Modular Muscle Coordination*. Cheung et al. [36] addressed how stroke affects muscle synergies for human upper limb movements. A group of stroke patients (31) with a wide range of unilateral motor impairment performed multiple upper-limb tasks using both unaffected (control) and affected arms, including seven virtual-reality tasks or a point-to-point reaching task in 3D space. During the task performance, EMG activities were collected from 17 shoulder, upper-arm, and forearm muscles: IS, TMaj, LD, RMaj, TU, PM, DELT_a_, DELT_m_, DELT_p_, TB, TB_l_, BB_l_, BB_m_, BR, BRD, SUP, and PRO. Nonnegative matrix factorization (NMF) identified muscle synergies necessary for an 80% *R*^2^ EMG reconstruction. Three distinct patterns of muscle synergies were identified, reflecting preservation, merging, and fractionation of the unaffected-arm muscle synergies in the stroke-affected arm, respectively. Furthermore, authors identified two or more synergies for the impaired arm as fractionations of one or multiple unaffected-arm synergies. Both merging and fractionation of synergies varied as a function of the severity of motor impairment. The findings were congruent with the idea that muscle synergies are structured in the spinal cord, and after cortical stroke, altered descending commands from supraspinal areas generate abnormal motor behaviours through faulty activations of the spinal modules. Overall, these results suggest that muscle synergies may be used as markers of the physiological status of stroke survivors.

In contrast to the rest of the existing literature on the topic of stroke upper-extremity muscle synergies that adopted motion or posture-oriented tasks, Roh et al. [38] examined the effects of stroke on the composition and recruitment of muscle synergies underlying the generation of isometric forces at the hand. Ten stroke survivors with severe impairment (FM score < 25/66; data collected from only impaired arm) and six age-matched control subjects (data collected from both arms) participated in the study. EMGs were recorded from shoulder and elbow muscles that include BRD, BB, TB_l_, TB_lat_, DELT_a_, DELT_m_, DELT_p_, and PM. By using the NMF algorithm, four synergies were identified. Of the four synergies, synergies with relatively isolated activation of the elbow flexors and extensors were conserved following stroke, respectively. However, the two synergies dominated by the activation of the shoulder muscles were altered. Structural changes in the synergies were consistent with an impaired ability to differentially activate the heads of deltoid. Recruitment of the altered shoulder muscle synergies was strongly associated with abnormal task performance, namely, a requisite rotation of the arm for lateral and upward force directions. The major findings suggest that stroke induces abnormal coordination of muscle activation in stroke survivors with severe impairment by altering the structure of muscle synergies that may contribute to poststroke deficits in the arm function.

*(3) Stroke: The Effects of Therapy on Muscle Synergies*. Hesam-Sariati et al. [42] investigated longitudinal changes in muscle synergies in stroke patients to determine if muscle synergy analysis could distinguish the level of impairment and the effect of therapy. The study cohort consisted of 24 stroke patients in a two-week intensive therapy, 13 of whom were available at the 6-month follow-up session. Each day, patients had 1 hour of formal therapy with an accredited exercise physiologist which was augmented by home practice using the Nintendo Wii-Sports game (Nintendo, Japan). To assess the motor coordination of the subjects, subjects played Wii-Baseball on the game system. Unlike previous studies in coordination, the studied task (simulated baseball swing) was primarily unconstrained. Surface EMG data were collected from 6 muscles on the affected side of the upper body: TM, DELT_m_, BB, ECR, FCR, and FID. The major results of the study were the following: (i) patients with low motor function had significantly fewer muscle synergies and significantly different distributions of synergy weightings and timings than patients with high motor function had, and (ii) the number of muscle synergies required to recreate the data did not change with time. The low motor function group had roughly three synergies compared to four for the high motor function group, suggesting that there was more complexity in the movements generated by the high motor function group. After training, all of the stroke subjects were able to make significant increases in the clinical motor-function assessments and the game performance in Wii-Baseball without increasing the number of required synergies. The reported findings allow affirming that the assessment of an increase in motor function has to be conducted by means of game performance rather than the use of muscle synergies since they remained unaltered after training.

*(4) Stroke: Neural Mechanism of Poststroke Muscle Coordination*. Godlove et al. [43] addressed how muscle synergies observed after stroke can be related to perilesional cortical activity. Subdural microelectrocorticography (ECoG) signals, recorded from the perilesional cortical area, and EMGs were simultaneously recorded in a stroke subject who suffered from refractory epilepsy and muscle weakness in the affected distal and proximal arm during horizontal reaching movements. Muscle activities were recorded from BRD, BB, TB_l_, TB_lat_, DELT_a_, DELT_m_, DELT_p_, and PM. To test the hypothesis that perilesional cortical activity is correlated with the activations of poststroke muscle synergies, NMF was applied to the EMG signal. A mix of both normal and abnormal resultant muscle synergies was surprisingly similar to the set of muscle synergies identified in a previous stroke study [38]. To quantify the cortical correlates of muscle synergy activation, they transformed each ECoG electrode's recorded signal into their component frequencies and power across time. Perilesional high-gamma oscillations, but no other frequency band, were significantly correlated with the activation of both normal and abnormal synergies. Considering the link between ECoG high-gamma activity and synchronized local neural spiking activity at the cortical surface [96, 97], the findings suggest that perilesional spiking may organize synergies after stroke.

*(5) Pain*. Research in both experimentally induced pain (Gizzi et al. [47] and Muceli et al. [48]) and chronic musculoskeletal pain (Heales et al. [49] and Manickaraj et al. [50]) shows that the presence of pain affects muscle recruitment in both the affected muscle and synergistic muscles during a task. In this section, we summarized the findings of Muceli et al. [48], who investigated muscle synergies in reaching a task in an induced pain paradigm, and Manickaraj et al. [50], who investigated muscle synergies in an isometric grip task in patients with chronic lateral epicondylalgia.

*(6) Pain: Reorganization of Muscle Synergies in Reaching due to Experimentally Induced Pain*. Muceli et al. [48] studied the changes in muscle synergies during a planar reaching task to determine if muscle activity remains modular in the presence of pain and if there are similarities between synergies in the pain and no-pain conditions. Eight healthy subjects had experimental muscle pain induced in the anterior deltoid muscle. In each of the four conditions (baseline, control injection, pain injection, and posttest), the subjects performed center-out- and out-center-reaching motions toward 12 evenly spaced targets. During the task, surface EMG data were collected from 12 muscles in the right arm: BRD, ANC, BB_l_, BB_lh_, BR, TB_l_, TB_lat_, PM, DELT_a,_ DELT_m,_ DELT_p_, and LD. The major results of the study were as follows: (i) muscle activity in the pain condition was modular; (ii) there was substantial shared information between the examined conditions; and (iii) the dynamic reorganization of the motor signals was subject-specific. In all four conditions, three synergies were required for adequate reconstruction of all subjects. In the pain condition, subjects showed at least two similar synergies from the baseline condition, but the shared synergies varied by subject. The study suggests that subjects recruited a new synergy as opposed to tuning the activation of a previous synergy in the presence of pain.

*(7) Pain: Quantification of Deficit in Grip Force for Patients with Lateral Epicondylalgia*. Manickaraj et al. [50] investigated forearm muscle synergies during a gripping task to determine whether the muscle coordination differed between chronic elbow pain patients and healthy subjects. The study cohort consisted of 11 chronic lateral epicondylalgia patients and a control group of age-, sex-, and limb-matched subjects. Each subject performed an isometric grip at 15% and 30% maximum voluntary contraction (MVC) in three different positions (neutral and 20° wrist flexion/extension). During each task, surface EMG data were collected from six muscles in the wrist and fingers: ECR, ECU, EDC, FCR, FCU, and FDS. The major result of the study was that there was a difference in the dimensionality of the synergies between lateral epicondylalgia patients and controls depending upon the magnitude of the grip force but not upon the position of the wrist. At lower levels of grip force, chronic elbow pain patients used only two synergies compared to the three synergies in the control group. The muscle synergies of the chronic elbow pain group had a greater similarity than those of the control group indicating less variability in the coordination. In most cases, the authors found no association between clinical measures and activation level of the muscle synergy. However, at the higher grip force, the pressure pain threshold was correlated with the activation level of the second (less dominant) synergy. The results open the possibility to use the number of synergy as an index to monitor the chronic elbow pain patients considering the difference of the number of synergies between healthy subjects and patients.

### 3.2. Robotics

We found 40 publications referencing muscle synergies for controlling robotic devices. Taking into account the inclusion criteria, we identified 11 papers for inclusion in this section.  Table 7 describes the distribution of the selected papers considering the specific robotic body segment.

8 papers, seven related to the robotic arm and one to the robotic hand, are deeply described in the following since they are the only papers that proposed a synergy-based control and that tested it by means of an experimental setup with robotic devices and subjects.

Details of all cited papers are reported in Table 8.

#### 3.2.1. Robotic Arm

Artemiadis et al. [52–56] have investigated how low-dimensional representations of sEMG signals of the upper limb can be used for the teleoperation of a robot arm in 3D space. Most of the studies were focused on the principal joints of the upper limb, that is, the shoulder and the elbow. A group of nine muscles that are mainly responsible for the studied motion was recorded: DELT_a_, DELT_p_, DELT_m_, PM, PM_c_, T, BB, BRD, and TB. Three able-bodied subjects were used (three males 27 ± 3 years old); during the experiment, the subjects were standing close to the robot arm, with their neck positioned looking at the front. The results of this study centered around two main issues. Firstly, the dimensionality reduction on the EMG signal was quite significant, since it not only revealed some interesting aspects regarding the 3D movements studied but also aided the matching between the EMG signals and motion since signal correlations were extracted, and the number of variables was drastically reduced. The latter led to the fact that a simple linear model with hidden states proved quite successful in mapping EMG signals to arm motion. The second important result of the study was that it was the first time a continuous profile of the 3D arm motion was extracted using only EMG signals. Most previous works extract only discrete information about motion, while there are some works that estimate continuous arm motion; however, they are constrained to isometric movements, single degrees of freedom (DOF), or very smooth motions. In conclusion, the findings of the studies raised the possibility to control a robot arm by reducing the dimensionality of the EMG signals, which is obtainable via muscle synergies.

Lunardini et al. [67] tested a method for controlling a robotic arm with 2 DOF; the control can reproduce the user's motion intention of the extension/flexion elbow and shoulder by using muscle synergies. The main novelty of the paper is the use of a higher number of involved muscles than in previous works, which used only one flexor muscle and one extensor muscle to control robotic arms. More specifically, recorded muscles were BRD, ANC, BB, TB, DELT_a_, DELT_m_, DELT_p_, and SS. Eight healthy subjects (age ranging from 24 to 33 years) were involved in the experimental protocol, which consisted in dynamic unconstrained flexion-extension movements of the elbow and the shoulder in the horizontal plane at different angles during isometric contractions. The entire protocol was repeated two times in two different days. Results showed that the proposed control is efficient in dynamic and isometric tasks and it is also repeatable over days. This study can represent a significant starting point toward the implementation of synergy-based myo-control for assistive robot devices.

#### 3.2.2. Robotic Hand

Ajoudani et al. [61] proposed an innovative synergy-based teleimpedance controller for a robotic hand, that is, the PISA-IIT SoftHand. The complexity of the hand movements was simplified by using motor patterns related to the first synergy, which is associated to the hand opening and closing. This synergy allows the activation of the FDS and the EDC that are the muscles used for grasp. The implemented teleimpedance controller permitted to grasp the hand taking into account the user's postural and synergy profiles in real time. The effectiveness of the novel control method was tested by enrolling two healthy subjects, who were asked to perform 20 grasping tasks at a varying cocontraction level. As reported by the authors, though the results need to be validated with amputees, the findings of this study allow affirming that synergy allows to grasp objects regardless of their elastic properties and to modify the compliance of the grasp via cocontraction.

### 3.3. Sports

70 papers were found by means of the electronic search in the above-mentioned databases. In addition, 23 further publications were identified using the cross-referencing evaluation. Applying the inclusion criteria, only 19 papers were reported in the present systematic review; in particular, Table 9 shows the distribution of these papers considering the sports involving the application of muscle synergy theory.

Details of all cited papers are reported in Table 10.

The following subparagraphs are organized based on the specific examined sport. In particular, three studies on cycling, two on rowing, and one on swimming, ice hockey, and fitness will be discussed since they are the only ones that proposed tangible outcomes useful for improving athlete performance assessment and/or planning athlete-specific training sessions.

#### 3.3.1. Cycling

In this section, three papers are deeply explained and discussed. The rationale of these papers is to provide useful information on muscle activity by means of muscle synergy theory to improve athlete performance and training.

Hug et al. [68] focused on understanding whether trained cyclists recruited the same muscle synergies regardless of the intersubject variability of EMG patterns. Through this scope, they enrolled nine male cyclists who were asked to pedal as long as possible at constant power equal to 80% of the maximum power output, specific for each subject. During the task, EMG signals were recorded from BF_lh_, GLU_max_, SM, VM, RF, VL, GM, GL, SOL, and TA. The authors found that three muscle synergies were sufficient to reconstruct EMG signals for all participants. Muscle synergy vectors and temporal activation patterns showed high mean correlation coefficients and low variance ratio values between subjects. These results can lead to the identification of a locomotor strategy common to the experienced cyclists; thus, recreational cyclists can train themselves to obtain similar muscle recruitment in order to increase their performance.

Turpin et al. [86, 87] investigated the advantage of using the standing position during intense cycling with respect to the seated position; in particular, they published two articles focusing on lower and upper limb muscles, respectively. Seventeen male untrained participants were involved in the studies to perform 10–12 s of pedalling in two positions, that is, seated and standing, and six power outputs, that is, 20%, 40%, 60%, 80%, 100%, and 120% of the power, corresponding to the spontaneous transition between the two positions computed in a preliminary test. Activity of 16 muscles was recorded; 9 lower limb muscles (TA, SOL, GM, VL, VM, RF, BF_lh_, GLU_max_, and SEMT) and 7 upper limb muscles (ES, LD, DELT_a_, TB, BB, BRD, and FD). Four and three muscle synergies were selected for lower and upper limb muscles, respectively. As regards lower limb muscles, the structure of muscle synergies was similar in the two positions, while differences were observed in the timing of extensor activation when the power output reached 600 W. Thus, authors suggested taking advantage of the standing position only when the power output is greater than 600 W. As concern upper limb muscles, a greater activation was observed in standing position; consequently, even though the upper limbs do not produce the power output, coaches and athletes should train interlimb coordination to improve athlete performance.

#### 3.3.2. Rowing

Two articles are resumed in the following to provide important suggestions about the training sessions of rowing.

Turpin et al. [70] designed an experimental study to verify if power outputs have effects on muscle synergies during rowing considering both untrained subjects and experienced rowers. Eight untrained male subjects and seven national-level male rowers were involved in the protocol that consisted in 2000 m rowing as fast as possible to individuate the mean power output and 2 min rowing at constant load corresponding to 60%, 90%, and 120% of the mean power output. The following muscles were recorded: TA, SOL, GL, GM, VL, VM, RF, BF, SEMT, GLU_max_, LD, ES, TM, BB, L, I, DELT_p_, TU, TL, TB, and FD. Three muscle synergies were selected for all participants in all power output conditions. Muscle synergy compositions, considering both the muscle synergy vectors and temporal activation patterns, were consistent across the three power outputs; however, a significant increase in the activation of TL and GLU_max_ was observed increasing the power output. In conclusion, the findings of this study suggest performing training sessions at lower power output with respect to the one used during an official event. Moreover, it should be efficient to further train the TL and GLU_max_ in terms of endurance force due to the greater activation with the increase of the power output.

Shaharudin and Agrawal [81] studied the robustness of the muscle synergies during incremental rowing VO_2max_, that is, the maximal oxygen consumption, since the aerobic capacity is one of the factors influencing rowing performance. Ten untrained male and ten collegiate male rowers were recruited. All participants performed an incremental ramp test starting at 25 W with increment of 25 W every 30 s. The task was interrupted for volitional exhaustion or if the power output was 10% below the set value for five consecutive rowing cycles. The right side of the body was sensorized applying surface EMG electrodes on SOL, GL, TA, BF_lh_, SEMT, RF, VL, ES, LD, TM, DELT_m_, TB, RA, PM, BB, and BRD. Three muscle synergies were sufficient to reconstruct EMG signals for both groups. Although similar temporal activation patterns were observed between the two groups, the strategy of the rower was completely different; in fact, they preferred to row slower with longer cycles. Significant differences on muscle coordination were found based on a different rowing economy. The results of the paper suggest optimizing the muscle coordination during the training stage in order to improve the performance in terms of rowing economy.

#### 3.3.3. Swimming

In this section, we reported the paper of Vaz et al. [82], since it provides useful guidelines for the training of beginner swimmers. Authors compared beginners and elite swimmers in terms of muscle coordination during breaststroke. Eight beginners and eight elites performed 25 m breaststroke at 100% of maximal effort without diving. Muscle patterns were acquired from 8 muscles (TB, BB, TI, PM. GM, TA, BF_lh_, and RF) on the right side of the body. Three muscle synergies were selected for thirteen out of sixteen participants, while four synergies were recruited for the remaining. No statistical differences were observed by comparing the Variability Account For computed with the subject-specific muscle synergy extraction and the one obtained after the cross-validation procedure; thus, it emerged that synergistic organization of muscle coordination during breaststroke does not depend on expertise. Conversely, four muscles of beginners were characterized by a negative shift in the activation time. From this last outcome, beginners could focus their training especially on the right timing of muscle activation to reach better performance.

#### 3.3.4. Ice Hockey

The paper reported in this section provides useful tools for the training of balance in ice hockey players. Kim et al. [84] studied the differences between elite athletes and nonathletes in terms of muscle activity in response to balance perturbations. Seven female elite athletes and seven female nonathletes were recruited; all participants were asked to stand on a moving platform and to maintain balance during ten trials of unexpected backward perturbation. Muscle activity was gathered from sixteen muscles, more specifically, SCM, EO, ESC, EST, ESL, ADD, RF, VM, TA, TFL, GLU_max_, GLU_med_, BF_lh_, PL, GM, and SOL. No significant differences in the number of muscle synergies were observed in both initial and reversal phases between the two examined groups; in the initial phase, muscle synergies ranged from four to eight and from five to six for elite players and nonathletes, respectively, while in the reversal phase, they ranged from six to eight in both groups. During the initial phase, two elite athletes' specific synergies, which were especially related to the control of the head, were found. This outcome could underline a specific ability to control balance when the head position is suddenly changed, as for example in brisk acceleration and deceleration skating. The specific synergies for the control of the head would be considered as a training strategy for female athletes to avoid potential risk of injury caused by external perturbation of the balance.

#### 3.3.5. Fitness

An article reported in this subparagraph provides an index suitable for the evaluation of the athlete performance in bench press exercise. Kristiansen et al. [77] focused their research on the comparison between power lifters and untrained subjects during bench press to understand if the training could influence the muscle synergy variability. They planned an experimental protocol with nine untrained and ten expert power lifters, who were asked to complete three sets of eight repetitions at 60% of the load that was previously computed during three repetitions at the maximum effort. Surface EMG were collected from nine muscles (PM, DELT_a_, BB, TB, LD, TI, ES, VL, and SOL) of the right side. Two muscle synergies were found for all the participants. However, the expert power lifters demonstrated less variability with respect to the untrained subjects in terms of both muscle synergy vectors and temporal activation patterns, especially during the concentric phase. Due to the obtained outcomes, the inner composition of the muscle synergies can be used as an index for the evaluation of the performance during bench press exercise. Moreover, such results could be interesting for personal trainers in order to customize training based on subjects' level of experience.

## 4. Conclusion

Understanding how the human brain generates neural commands to control muscles during motor tasks still arouses great interest; in the last decades, the factorization of the EMG signals by means of muscle synergies has been proposed in order to understand the neurophysiological mechanisms related to the central nervous system ability in reducing the dimensionality of the muscle control.

This review gives to research groups involved in muscle synergies an overview on tangible applications of this theory in clinics, robotics, and sports. This offers the possibility to inspire new ideas for future works in muscle synergy fields.

## Figures and Tables

**Table 1:** Criteria for quality assessment of internal validity (IV), statistical validity (SV), and external validity (EV).

Criteria	Possible outcomes
*Aim of the work*	
(1) Description of a specific, clearly stated purpose (IV)	+/−
(2) The research question is scientifically relevant (EV)	+/−
*Inclusion criteria (selection bias)*	
(3) Description of inclusion and exclusion criteria (IV-EV)	+/−
(4) Inclusion and exclusion criteria are the same for all tested groups (IV)	+/−
(5) Inclusion and exclusion criteria reflect the general population (EV)	+/−
*Data collection (performance bias)*	
(6) Data collection is clearly described and reliable (IV-EV)	+/−
(7) Same data collection method used for all the subjects (IV)	+/−
(8) Data collection reflects the usual methodology in the field (EV)	+/−
*Data loss (attrition bias)*	
(9) Different data loss between groups (IV)	+/−
(10) Data loss < 20% (EV)	+/−
*Outcome (detection bias)*	
(11) Outcomes are topic-relevant (EV)	+/−
(12) Outcomes are the same for all the subjects (IV)	+/−
*Data presentation*	
(13) Frequencies of most important outcome measures (IV)	+/−
(14) Presentation of the data is sufficient to assess the adequacy of the analysis (IV)	+/−
*Statistical approach*	
(15) Appropriate statistical analysis techniques (SV)	+/−
(16) Clearly state the statistical test used (SV)	+/−
(17) State and reference the analytical software used (SV)	+/−
(18) Sufficient number of subjects (SV)	+/−

**Table 2:** Acronyms of human full-body muscles.

Acronym	Muscle
ADD	Adductor magnus
AL	Adductor longus
ANC	Anconeus
BB	Biceps brachii
BB_m_	Biceps brachii (medius)
BB_l_	Biceps brachii (long head)
BF_lh_	Biceps femoris (long head)
BR	Brachialis
BRD	Brachioradialis
DELT_a_	Deltoideus (anterior)
DELT_m_	Deltoideus (medius)
DELT_p_	Deltoideus (posterior)
ECR	Extensor carpi radialis
EDC	Extensor digitorum communis
ECU	Extensor carpi ulnaris
EDL	Extensor digitorum longus
EO	External oblique
ES	Erector spinae
ESC	Erector spinae cervical region
ESL	Erector spinae lumbar region
EST	Erector spinae thoracic region
FCR	Flexor carpi radialis
FCU	Flexor carpi ulnaris
FD	Flexor digitorum
FDI	First dorsal interosseous
FDS	Flexor digitorum superficialis
GL	Gastrocnemius lateralis
GLU_max_	Gluteus maximus
GLU_med_	Gluteus medius
GM	Gastrocnemius medialis
I	Iliocostalis
IS	Infraspinatus
L	Longissimus
LD	Latissimus dorsi
PL	Peroneus longus
PM	Pectoralis major
PM_c_	Pectoralis major (clavicular)
PRO	Pronator teres
RA	Rectus abdominis
RF	Rectus femoris
RMaj	Rhomboid major
SCM	Sternocleidomastoid
SEMT	Semitendinosus
SM	Semimembranosus
SOL	Soleus
SS	Supraspinatus
SUP	Supinator
T	Trapezius
TA	Tibialis anterior
TB	Triceps brachii
TB_l_	Triceps brachii (long head)
TB_lat_	Triceps (lateral head)
TFL	Tensor fasciae latae
TI	Trapezius inferior
TM	Trapezius medius
TMaj	Teres major
TU	Trapezius upper
VL	Vastus lateralis
VM	Vastus medialis

**Table 3:** Papers using muscle synergies in clinical practice/rehabilitation for locomotion and balance task.

	*N*	%	References
Stroke	7	44.43%	[17–20, 22–24]
Cerebral palsy (CP)	3	16.67%	[25, 27, 51]
Spinal cord injury (SCI)	2	11.11%	[28, 29]
Parkinson's disease (PD)	2	11.11%	[30, 31]
Multiple sclerosis	1	5.56%	[32]
ACL^1^ injury	1	5.56%	[33]
Aging	1	5.56%	[34]

^1^Anterior cruciate ligament.

**Table 4:** Selected locomotion and balance studies using muscle synergies.

Reference	Pathology	Aim	Subjects	Tasks	Muscles (segments)	Outcomes
[17] Clark et al. 2010	Stroke	Evaluate change in motor control	55 patients 20 healthy subjects	Overground walking and treadmill	16 (lower limbs)	(i) Reduced synergies in patients (ii) Number of synergies related to walking performance
[18] Ferrante et al. 2016	Stroke	Design a multichannel functional electrical stimulation controller	2 patients 13 healthy subjects	Overground walking and treadmill	7 (lower limb)	(i) Four synergies in healthy subjects (ii) Three synergies in patients (iii) Four synergies after FES
[19] Kautz et al. 2017	Stroke	Evaluate change in motor control	56 patients 17 healthy subjects	Overground walking and treadmill	16 (lower limbs)	(i) Similar muscle synergies in the two walking conditions
[20] Gizzi et al. 2011	Stroke	Evaluate change in motor control	10 patients 10 healthy subjects	6 m long walking	32 (full-body)	(i) Different muscle synergy vectors (ii) Similar activation profiles and number of synergies
[22] Hashiguchi et al. 2016	Stroke	Evaluate change in muscle synergies due to rehabilitation	13 patients	Overground walking	8 (lower limb)	(i) Same number of muscle synergies (ii) Merging synergies as an index for motor coordination
[23] Coscia et al. 2015	Stroke	Assess the relationship between gait asymmetry and muscle synergies	12 patients 10 healthy subjects	Walking on a treadmill	24 (lower limbs)	(i) Similar muscle synergies in less affected limb (ii) Altered muscle synergy organization in the affected limb
[24] Barroso et al. 2017	Stroke	Assess the walking performance	9 patients	Overground walking	22 (trunk and lower limb)	(i) Fewer synergies in the paretic side (ii) Muscle synergies useful for walking performance assessment
[25] Steele et al. 2015	Cerebral palsy	Examine motor modification	549 patients 84 healthy children	Overground walking	5 (lower limb)	(i) Reduced synergies in patients (ii) Number of synergies as index for CP assessment
[27] Shuman et al. 2017	Cerebral palsy	Evaluate the repeatability of muscle synergies across days	5 patients 6 healthy children	Overground walking	16 (lower limbs)	(i) Reduced synergies in patients (ii) Muscle synergies repeatable between days in both groups
[28] Fox et al. 2013	Spinal cord injury	Evaluate change in motor control	5 patients 5 healthy children	Locomotion tasks	12 (lower limb)	(i) Reduced synergies in patients (ii) Similar modular organization for all tested tasks
[29] Hayes et al. 2014	Spinal cord injury	Quantify neuromuscular deficits in muscle coordination	8 patients 8 healthy subjects	Overground walking	14 (lower limb)	(i) Different synergy organization (ii) Similar synergy during walking with metronome
[30] Allen et al. 2017	Parkinson's disease	Assess changes in control of gait and balance after dance-based rehabilitation	9 patients	Dance (tango)	13 (lower back and limb)	(i) No modifications in muscle synergies (ii) Decrease in muscle synergy variability
[31] Rodriguez et al. 2013	Parkinson's disease	Evaluate change in motor control	15 patients 14 healthy older	Walking on a treadmill	16 (lower limbs)	(i) Reduced synergies in patients (ii) Similar muscle synergies (iii) Different activation profiles
[32] Lencioni 2016	Multiple sclerosis	Evaluate change in motor control	17 patients 12 healthy subjects	Overground walking	8 (lower limb)	(i) Similar muscle synergy organization (ii) Different activation profiles
[33] Serrancolì et al. 2016	Anterior cruciate ligament	Evaluate change in motor control	18 patients 10 healthy subjects	Overground walking	16 (lower limbs)	(i) Higher cocontraction in patients (ii) Different activation profiles
[34] Monaco et al. 2010	Aging	Evaluate change in motor control	7 younger subjects 7 older subjects	Overground walking	12 (lower limb)	(i) Similar muscle synergy organization
[51] Tang et al. 2015	Cerebral palsy	Assess lower extremity dysfunction	12 patients 8 healthy children 10 healthy adults	Overground walking	16 (lower limbs)	(i) Greater variability in muscle synergy organization in patients (ii) Relationship between muscle synergies and motor dysfunctions

**Table 5:** Papers using muscle synergies in clinical practice/rehabilitation specific to the upper body.

	*N*	%	References
Stroke	9	61.10%	[36–44]
Cerebral palsy	1	5.56%	[26]
Spinal cord injury (SCI)	1	5.56%	[45]
Dystonia	1	5.56%	[46]
Pain	4	22.22%	[47–50]

**Table 6:** Selected upper limb functionality studies using muscle synergies.

Reference	Pathology	Aim	Subjects	Tasks	Muscles (segments)	Outcomes
[26] Tang et al. 2017	Cerebral palsy	Assess the upper limb motor dysfunction	14 patients 10 healthy children	Reaching tasks	10 (trunk and upper limb)	(i) Reduced synergies in patients (ii) Greater synergy variability in patients
[36] Cheung et al. 2012	Stroke	Assess the upper limb motor dysfunction	31 patients	Reaching tasks	17 (upper limbs)	(i) Reduced synergies in affected arm (ii) Muscle synergies as marker for physiological evaluation
[37] Lee et al. 2013	Stroke	Assess the ability to modulate muscle coordination	14 patients 4 healthy subjects	Isometric tasks	9 (upper limb)	(i) Correlation with clinical scores (ii) Muscle synergy vectors as index for pathology severity
[38] Roh et al. 2013	Stroke	Assess the upper limb motor dysfunction	10 patients 6 healthy subjects	Isometric tasks	8 (upper limb)	(i) Different muscle synergy organization for shoulder muscle
[39] Tropea et al. 2013	Stroke	Assess changes in motor control after neurorehabilitation	6 patients 10 healthy subjects	Reaching task	10 (upper limb)	(i) Different muscle synergy organization for shoulder muscle (ii) No difference on muscle synergy organization after rehabilitation
[40] Garcia-Cossio et al. 2014	Stroke	Characterize motor control	33 patients	Movements of arm and hand	8 (upper limb)	(i) Correlation between muscle synergies and hand movement functionality
[41] Roh et al. 2015	Stroke	Assess the upper limb motor dysfunction	16 patients 10 healthy subjects	Isometric tasks	8 (upper limb)	(i) Four synergies in both groups (ii) Different muscle synergy organization for shoulder muscle
[42] Hesam-Shariati et al. 2017	Stroke	Use synergies as severity index	24 patients	Wii-Baseball game	6 (upper limb)	(i) Reduced synergies in patients with high pathology severity
[43] Godlove et al. 2016	Stroke	Assess changes in motor control	1 patient	Reaching tasks	8 (upper limb)	(i) Abnormal muscle synergy organization and cortical activation in the stroke patient
[44] Li et al. 2017	Stroke	Define metrics for assessing motor functions	10 patients 9 healthy subjects	Reaching tasks	7 (upper limb)	(i) Different muscle synergy organization in patients
[45] Zariffa et al. 2012	Spinal cord injury	Examine change in motor control	8 patients 10 healthy subjects	Gripping tasks	8 (hand)	(i) Different synergies in patients (ii) No correlation with motor ability
[46] Lunardini et al. 2017	Dystonia	Examine change in motor control	9 patients 9 healthy children	Writing tasks	8 (upper limb)	(i) Similar muscle synergy organization between groups
[47] Gizzi et al. 2015	Pain	Examine change in neck motor control	8 healthy subjects with pain induced	Reaching tasks	12 (trunk and neck)	(i) Different synergy organization with and without induced pain
[48] Muceli et al. 2014	Pain	Examine change in motor control	8 healthy subjects with pain induced	Reaching tasks	12 (upper limb)	(i) Higher variability of muscle synergy organization with pain
[49] Heales et al. 2016	Lateral epicondylalgia	Examine change in coordination of forearm	20 patients 14 healthy patients	Gripping tasks	6 (upper limb)	(i) Two synergies in both groups (ii) Different muscle synergy organization between groups
[50] Manickaraj et al. 2017	Lateral epicondylalgia	Examine change in motor coordination	11 patients 11 healthy subjects	Gripping tasks	6 (hand)	(i) Reduced synergies in patients (ii) Use of muscle synergies as index to monitor pain

**Table 7:** Papers using muscle synergies in robotics.

	*N*	%	References
Arm	8	62.50%	[52–56, 62, 64, 67]
Hand	1	18.75%	[61]
Leg	2	18.75%	[65, 66]

**Table 8:** Selected robotic studies using muscle synergies.

Reference	Robot	Aim	Subjects	Tasks	Muscles (segment)	Outcomes
[52–56] Artemiadis et al. 2006–2012	Arm	Investigate the use of low-dimensional EMG for teleoperation of robot	3 healthy subjects	3D-reaching task	9 (upper limb)	(i) Feasible robot control with significant EMG data reduction (ii) Possible control in 3D space
[61] Ajoudani et al. 2013	Hand	Propose innovative synergy-based controller	2 healthy subjects	Grasping task	2 (hand)	(i) Synergy-based control able to perform grasping (ii) Synergy-based control adaptable to grasp compliance
[62] Ison et al. 2015	Arm	Propose an alternative approach for long-term myoelectric control	8 healthy subjects	Reaching task	2 (upper limb)	(i) Differences in muscle synergy organization do not influence task performance
[64] Cimolato et al. 2017	Arm	Describe a muscle synergy-based control	1 healthy subject	Reaching task	16 (trunk and upper limb)	(i) Four synergies to control robot motion (ii) Performance improvement with synergy-based control
[65] Cunha et al. 2016	Leg	Describe a muscle synergies based control	Simulation	Overground walking	8 (lower limbs)	(i) Two synergies to control six joints (ii) Muscle synergies robust across strides and speeds
[66] Watanabe et al. 2016	Leg	Propose innovative synergy-based controller	5 healthy subjects	Pedalling	16 (lower limbs)	(i) Three synergies to control musculoskeletal robot
[67] Lunardini et al. 2016	Arm	Propose innovative synergy-based controller	8 healthy subjects	Isometric contractions	8 (lower limb)	(i) Repeatable control across days (ii) Robust synergy-based control for arm robot

**Table 9:** Papers using muscle synergies in sports.

	*N*	%	References
Cycling	5	30.00%	[68, 69, 74, 86, 87]
Rowing	3	15.00%	[70, 71, 81]
Swimming	1	5.00%	[82]
Ice hockey	1	5.00%	[84]
Fitness	5	25.00%	[77–80, 83]
Athletics	2	10.00%	[73, 85]
Football	1	5.00%	[76]
Artistic gymnastics	1	5.00%	[72]

**Table 10:** Selected sport studies using muscle synergies.

Reference	Sport	Aim	Subjects	Tasks	Muscles (segment)	Outcomes
[68] Hug et al. 2010	Cycling	Examine muscle synergy variability	9 cyclists	Pedalling	10 (lower limb)	(i) Similar muscle synergy vectors (ii) Similar activation profiles
[69] Hug et al. 2011	Cycling	Evaluate the effect of movement mechanics	11 cyclists	Pedalling	11 (lower limb)	(i) Training can be performed at low power output
[70] Turpin et al. 2011	Rowing	Verify the effects of power output	7 rowers 8 untrained subjects	2000 m rowing	21 (full-body)	(i) Similar muscle synergies were found in the two walking conditions
[71] Turpin et al. 2011	Rowing	Evaluate the effect of expertise	7 rowers 8 untrained subjects	2-min rowing	23 (full-body)	(i) Similar activation profiles between groups (ii) Different muscle synergy vectors between groups
[72] Frère and Hug 2012	Artistic gymnastics	Examine muscle synergy variability	9 gymnasts	Backward giant swings	12 (full-body)	(i) Three synergies for all subjects (ii) Difference in the third synergy
[73] Frère et al. 2012	Athletics	Evaluate the catapult effect on maximum height	7 vaulters	10 pole vaults	10 (upper limbs)	(i) Similar activation profiles (ii) Different muscle synergy vectors (iii) No influence on maximum height
[74] De Marchis et al. 2013	Cycling	Investigate the muscle coordination	9 untrained subjects	Pedalling	8 (lower limb)	(i) More synergies with respect to expertise (ii) Variability of activation profiles (iii) Similarity of muscle synergy vectors
[76] Cruz Ruiz et al. 2015	Football	Design controllers for avatar animation	1 footballer	Right-hand throws	16 (trunk and upper limb)	(i) Three synergies for all throws (ii) Repeatable and robust synergies
[77] Kristiansen et al. 2015	Fitness	Evaluate the effect of expertise	10 lifters 9 untrained subjects	Bench press	9 (full-body)	(i) Less variability in expert (ii) Use composition of muscle synergies as performance index
[78] Kristiansen et al. 2016	Fitness	Assess change in motor control after a 5-week training	30 untrained subjects	Bench press	13 (full-body)	(i) No difference after training (ii) Higher maximum load after training
[79] Smale et al. 2016	Fitness	Verify the effect of fatigue	9 healthy subjects	Two-leg squatting	12 (lower limb)	(i) Difference in muscle synergy composition after fatigue (ii) Muscle synergy as index of fatigue
[80] Kristiansen et al. 2016	Fitness	Evaluate the between-day reliability	21 healthy subjects	Bench press	13 (full-body)	(i) Same muscle synergy organization across days
[81] Shaharudin and Agrawal 2016	Rowing	Evaluate the effect of incremental power output	10 rowers 10 untrained subjects	Rowing	16 (full-body)	(i) Similar activation profiles between groups (ii) Muscle coordination influence rowing economy
[82] Vaz et al. 2016	Swimming	Evaluate the effect of expertise	8 swimmers 8 beginners	25 m breaststroke	8 (full-body)	(i) No different muscle synergy organization between groups (ii) Different activation time in expert
[83] Chen et al. 2017	Fitness	Investigate the intra- and interlimb muscle coordination	20 healthy subjects	Crawling	32 (full-body)	(i) Two synergies for both limbs (ii) Different muscle synergy vectors among speeds
[84] Kim et al. 2017	Ice hockey	Evaluate the response to balance perturbation	7 expert 7 untrained subjects	Balance perturbation	16 (full-body)	(i) Specific synergy to control head movement in expert
[85] Nishida et al. 2017	Athletics	Evaluate the effect of strike patterns	10 healthy subjects	Running	12 (trunk and lower limbs)	(i) Different synergy vectors, timing, and duration of activation profiles
[86] Turpin et al. 2017	Cycling	Evaluate the effect of seating and standing position	17 untrained subjects	Pedalling	9 (lower limb)	(i) Four synergies for all subjects (ii) Similar muscle synergy organization between positions
[87] Turpin et al. 2017	Cycling	Evaluate the effect of seating and standing position	17 untrained subjects	Pedalling	7 (trunk and upper limb)	(i) Three synergies for all subjects (ii) Greater activation during standing position
